# Mapping the complete glycoproteome of virion-derived HIV-1 gp120 provides insights into broadly neutralizing antibody binding

**DOI:** 10.1038/srep32956

**Published:** 2016-09-08

**Authors:** Maria Panico, Laura Bouché, Daniel Binet, Michael-John O’Connor, Dinah Rahman, Poh-Choo Pang, Kevin Canis, Simon J. North, Ronald C. Desrosiers, Elena Chertova, Brandon F. Keele, Julian W. Bess, Jeffrey D. Lifson, Stuart M. Haslam, Anne Dell, Howard R. Morris

**Affiliations:** 1 Department of Life Sciences, Imperial College London, South Kensington Campus, London, SW7 2AZ, UK; 2 BioPharmaSpec, Suite 3.1 Lido Medical Centre, St. Saviours Road, Jersey, JE2 7LA, UK; 3 Department of Pathology, University of Miami, Miami, Florida, 33136, USA; 4 AIDS and Cancer Virus Program, Leidos Biomedical Research, Inc., Frederick National Laboratory for Cancer Research, Frederick, Maryland 21702, USA

## Abstract

The surface envelope glycoprotein (SU) of Human immunodeficiency virus type 1 (HIV-1), gp120^SU^ plays an essential role in virus binding to target CD4+ T-cells and is a major vaccine target. Gp120 has remarkably high levels of N-linked glycosylation and there is considerable evidence that this “glycan shield” can help protect the virus from antibody-mediated neutralization. In recent years, however, it has become clear that gp120 glycosylation can also be included in the targets of recognition by some of the most potent broadly neutralizing antibodies. Knowing the site-specific glycosylation of gp120 can facilitate the rational design of glycopeptide antigens for HIV vaccine development. While most prior studies have focused on glycan analysis of recombinant forms of gp120, here we report the first systematic glycosylation site analysis of gp120 derived from virions produced by infected T lymphoid cells and show that a single site is exclusively substituted with complex glycans. These results should help guide the design of vaccine immunogens.

The envelope glycoprotein spikes on HIV-1 virions are comprised of trimers of non-covalently associated gp120^SU^/gp41^TM^ (transmembrane envelope protein, TM–abbreviations are defined in Supplementary Table S16) heterodimers which are produced by furin-mediated proteolytic cleavage of the gp160 glycoprotein precursor. The HIV-1 envelope glycoprotein (Env) has remarkable levels of N-linked glycosylation with about 50% of its mass being glycan-derived. This extensive glycosylation constitutes a “glycan shield” which helps to protect the virus from antibody-mediated neutralization. However, with the isolation and detailed characterization of multiple broadly neutralizing monoclonal antibodies (bnAbs) in recent years, it has become clear that the glycans themselves can be involved in Env recognition by such antibodies. Indeed, the glycans on gp120^SU^, which is the more densely glycosylated component of the heterodimer, appear to be essential constituents of the binding sites for some of the most potent of these bnAbs.

Depending on the isolate, gp120^SU^ has about 25 N-glycosylation sites, many of which are clustered within, or in close proximity to variable domains of the protein. Two of the best characterized bnAbs, PG9 and PGT128, target glycans associated with the variable regions V1/V2 and V3, respectively^1^,^2^. Much is known about the glycosylation of a great many gp120 variants expressed using recombinant methods in a variety of cell lines^3^,^4^,^5^,^6^,^7^. Thus, it has been shown that recombinant gp120 (rgp120) is rich in both complex-type and oligomannose N-glycans, with the former predominating. For example, early work on rgp120 from isolate HIV-1_IIIB_, expressed in Chinese hamster ovary (CHO) cells as a truncated, secreted product, identified 24 occupied sites, 13 of which were substituted with complex glycans whilst 11 sites were mainly oligomannose^6^. More recently it has been shown that the glycosylation profile can differ substantially, depending on the host-cells from which the recombinant gp120 is produced^7^. Nonetheless, the high abundance of complex-type glycans in rgp120 is preserved, irrespective of the host cell. This is in sharp contrast to what has been found for virion-derived gp120^SU^ where glycan profiling experiments have shown that the oligomannose content varies substantially depending on the strain, and can constitute up to 80% of the glycome^8^,^9^. High levels of oligomannose have also recently been found in HIV-1 envelope glycoprotein when expressed recombinantly as membrane anchored^10^ or soluble trimers^11^,^12^. In previous virion studies, limitations in sample availability precluded systematic site-specific glycan analysis. Thus only the global glycan content was determined. Consequently the site occupancy knowledge gained from analysing recombinant gp120^SU^ has not so far been compared with that from virion derived gp120.

Defining site specific glycosylation on the virion envelope-glycoprotein should facilitate the rational design of glycopeptide antigens as targets for HIV vaccine development. Fortunately, progress in deriving cell lines that produce HIV-1 particles with increased gp120 content and methods for purifying gp120 from virions, coupled with improvements in glycoproteomic technologies, means that defining site occupancy, although very challenging, is now a feasible goal. Here we report our systematic glycoproteomic investigation of site-specific N-glycosylation of gp120 purified from HIV-1 virions produced by an infected T lymphoid cell line. We show that 20 of the 24 glycosylation sites in the gp120 are almost exclusively occupied with oligomannose glycans, two sites are a mixture of complex and hybrid glycans, one site carries a mixture of similar quantities of all three glycan classes, and one site is exclusively substituted with complex glycans. The latter is located in the V1 domain. Based on research on other HIV strains, this site is likely to be important for binding by the peptidoglycan (PGT) family of potent bnAbs.

## Results

### Production and purification of HIV-1 BaL/SUPT1-R5 Env for site-specific glycoanalysis of gp120

**Figure 1:**
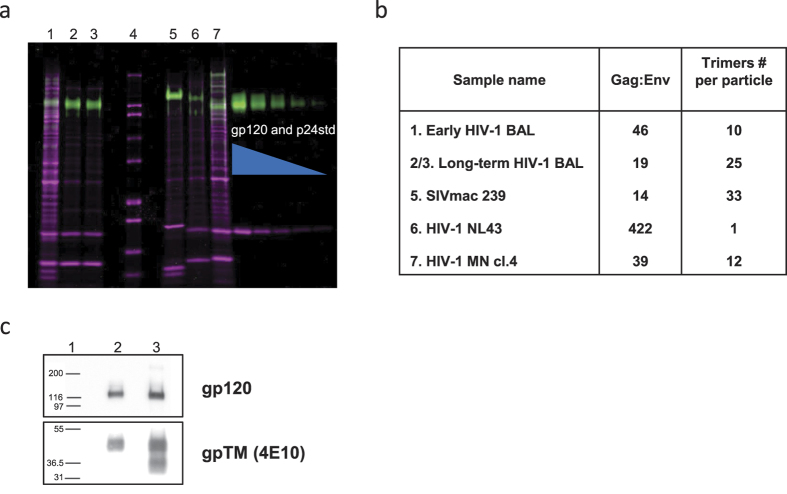
Virion-associated Env analysis. **(a)** A two-dye SYPRO-stained (total protein magenta, glycoproteins green) SDS-PAGE gel analysis of early (1) vs. long-term culture (2,3) of HIV-1_BaL_. Early HIV-1_BaL_ was prepared from a short term passage culture using virus originally obtained from the NIH AIDS Research and Reference Reagent Program. Long-term samples were derived from the original cultures, after extended culture. Gel was calibrated with the gp120^SU^ (200–12.5 ng) and purified p24^CA^ (600–37.5 ng) standards. MW standards are found in lane 4. Positive control samples are found in lanes 5–7; SIV_mac239_,(5), HIV-1_NL43_ (6), HIV-1_MN_ (7). **(b)** Table shows calculated Gag:Env ratios for the different virus preparations based on densitometric measurements normalized for molecular weights, and estimated average trimers per virion, assuming 1400 gag molecules per particle^14^. **(c)** mAb 4E10 anti-HIV-1 TM immunoblot analysis of early (2) vs. long-term (3) HIV-1BaL culture series; 1 is MW standards. Short term passage virus shows only full length TM. In contrast, long-term cultured virus shows a mixture of TM species including both full length gp41 and truncated ~gp36.

Previously, it was found that HIV-1 and simian immunodeficiency virus (SIV) virions, produced from various T-cell lines contain a calculated average of between 7 and 14 envelope glycoprotein (Env) trimers per virion^13^,^14^. We have now performed biological, molecular, and structural analysis of human immunodeficiency virus (HIV-1) virions produced from *in vitro* propagation in SUPT1-CCR5 cells. SDS-PAGE, immunoblot analysis and sequence analysis were used to characterize viral proteins. Gag (group antigens) and Env content were monitored with a sensitive, specific, calibrated fluorescent dye staining technique^15^. Virus was produced by inoculating SUPT1-CCR5 cells with HIV-1_BaL_ obtained from the National Institutes of Health (NIH) AIDS Reagent Program. Following an initial cytopathic crisis, a long term outgrowth from the infected culture produced large amounts of virus, without evident cytopathology, associated with loss of CD4 expression on producing cells. Env sequence analysis of the virus produced by a cell line derived from this outgrowth culture, designated HIV1 BaL/SUPT1-R5 [CLN204], revealed a substantially homogeneous virus population and identified changes, including truncations in the transmembrane envelope protein that were associated with increased virion Env content (Fig. 1a,b). Determination of the Gag/Env ratio for viral samples harvested from early and late cultures was performed by fluorescence-based quantitation^15^. A merged image of a representative gel is presented in Fig. 1a. The masses of HIV-1 capsid protein p24 (p24^CA^) and gp120^SU^ in the virion samples were then calculated by interpolation of integrated pixel densities for the test samples onto standard curves obtained from a dilution series of calibrated protein standards. Immunoblot analysis of early vs. long-term culture supernatants confirmed the presence of a truncated form of TM (~gp36) in the late culture compared to viral sample harvested early (only gp41) (Fig. 1c). This virus retained robust infectivity in both single cycle (TZM-bl cells) and spreading infection (SUPT1-R5 cells) and demonstrated X4/R5 dual tropism. Of note, a similar 100-amino acid truncation in the cytoplasmic tail of gp41 in HIV-1_RF_ was described previously after *in vitro* passage^16^. While increased virion Env content^13^,^17^,^18^,^19^ with maintenance of infectivity is a well described phenomenon for SIVs with certain truncations in TM, it has been more challenging to derive robustly infectious HIV-1 viruses with increased virion Env content using similar truncations^13^,^20^,^21^.

### HIV-1_BaL_ gp120 purification for glycoanalysis

**Figure 2:**
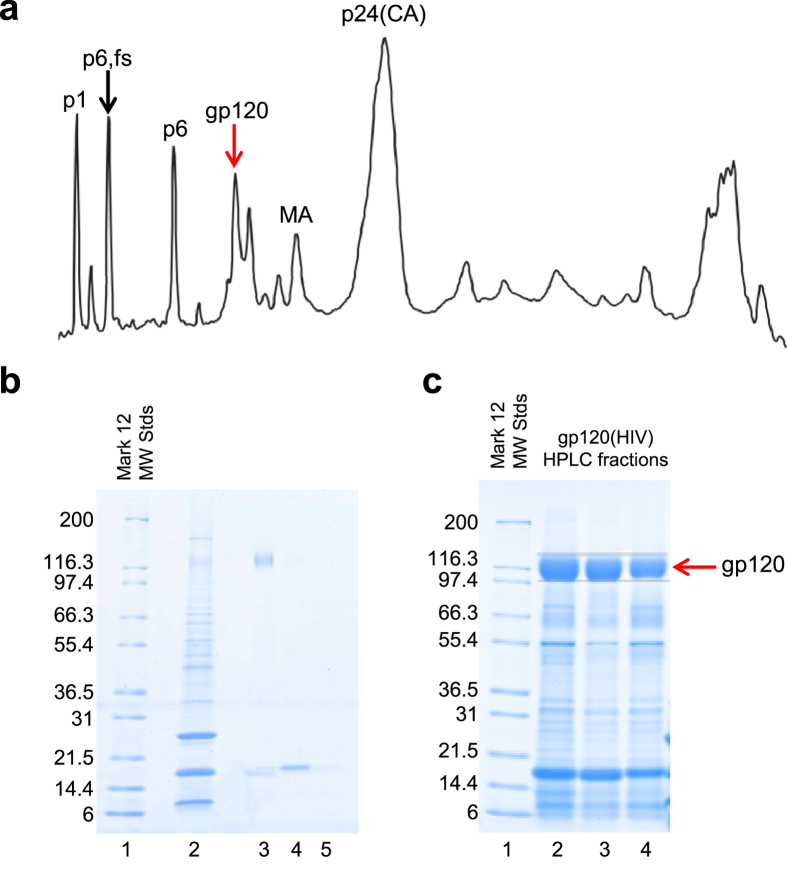
HPLC fractionation of HIV-1 BaL/SupT1-R5 used to purify gp120. **(a)** HPLC was used to separate HIV-1 BaL/SUPT1-R5 after inactivation with aldrithiol-2^50^,^51^,^52^ under non-reducing conditions using 206nm UV absorbance to detect proteins. Viral protein peaks (identified by SDS-PAGE gels, sequencing and immunoblot analysis) are labelled above the chromatograph. **(b)** Coomassie blue-stain of SDS-PAGE gel was used to analyse gp120^SU^ corresponding fractions, with the molecular mass of standards denoted (Lane 1). Lane 2 represents pre-fractionated, long term cultured virus (1μL). We analysed HPLC fractions (Lanes 3–5). **(c)** Coomassie blue stained SDS-PAGE gel following HPLC purification of gp120^SU^ (lanes 2–4). Lanes 1 represent MW standards. The band containing gp120^SU^ was excised for glycomic/gylcoproteomic analyses.

Virions derived from the HIV-1 BaL/SUPT1-R5 cell line [CLN204] were density gradient-purified and the virion-associated gp120 was partially purified by HPLC (Fig. 2). The HPLC fractions containing gp120 were then loaded onto a SDS-PAGE gel and gp120 was purified. After destaining, the gp120–containing bands were excised and transferred to tubes containing a 1% (v/v) acetic acid (aq.) solution. Approximately 260 μg of gp120^SU^ derived from virions produced by the HIV-1 BaL/SUPT1-R5 cell line [CLN204] was purified for the purposes of these analyses.

### Strategy for defining gp120 glycosylation

**Figure 3:**
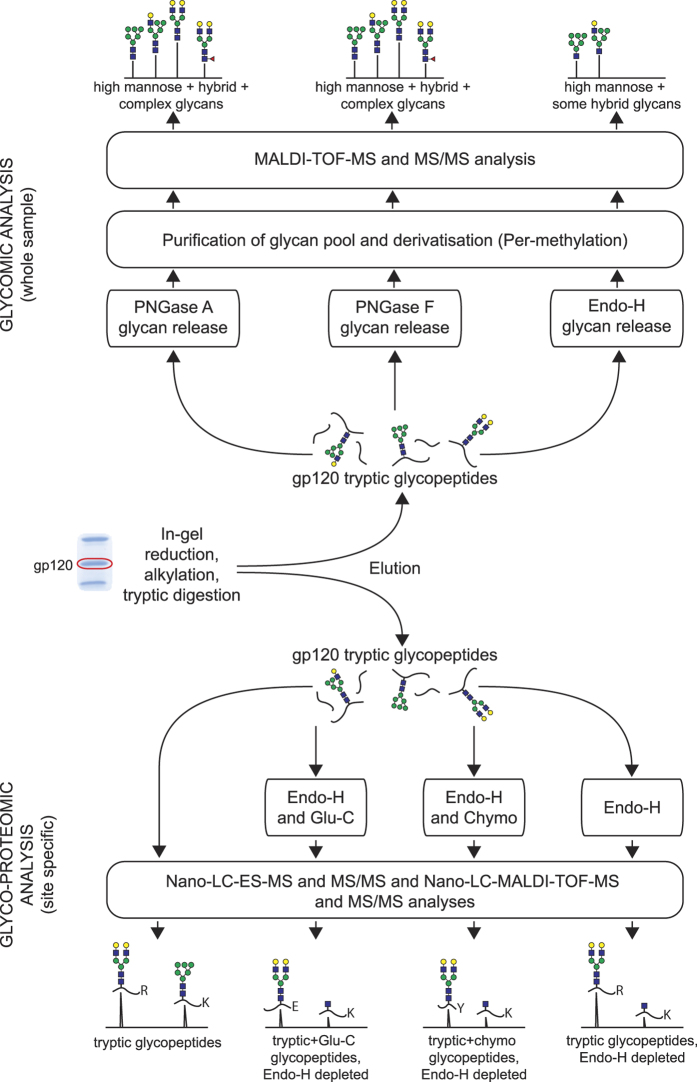
Simplified workflow of the glycomic and glycoproteomic methodologies employed. The samples, in the form of isolated gp120 gel bands, were excised and proteolytically digested in-gel. The resultant peptides/glycopeptides were then eluted and subjected to either a detailed glycomic workflow (upper half of the figure) or in-depth glycoproteomic analyses (lower half of the figure).

Mapping of all 24 N-glycosylation sites in gp120 was achieved by performing multiple glycomic and glycoproteomic analyses, each employing low microgram quantities of sample. Acknowledging the limitations inherent in each of the different techniques we utilized, we employed multiple different, complementary methods to obtain a more comprehensive analysis. In the glycomic experiments, glycans were released from tryptic digests by PNGase F, PNGase A or Endoglycosidase H (Endo H) and were permethylated prior to matrix-assisted laser desorption ionization tandem time of flight mass spectrometry (MALDI-TOF-TOF MS and MS/MS) analysis. Glycoproteomic analyses exploited both off-line and on-line nano-liquid chromatography (LC)-MS/MS, with MALDI and electrospray (ES) ionisation, respectively. Samples for glycoproteomics included: (a) tryptic digests; (b) sequential tryptic and Endo H digests; (c) sequential tryptic, Endo H and chymotryptic digests; (d) tryptic, Endo H and Endoproteinase GluC (Glu-C) digests. The overall strategy is depicted in Fig. 3.

### The majority of gp120 glycans are oligomannose

**Figure 4:**
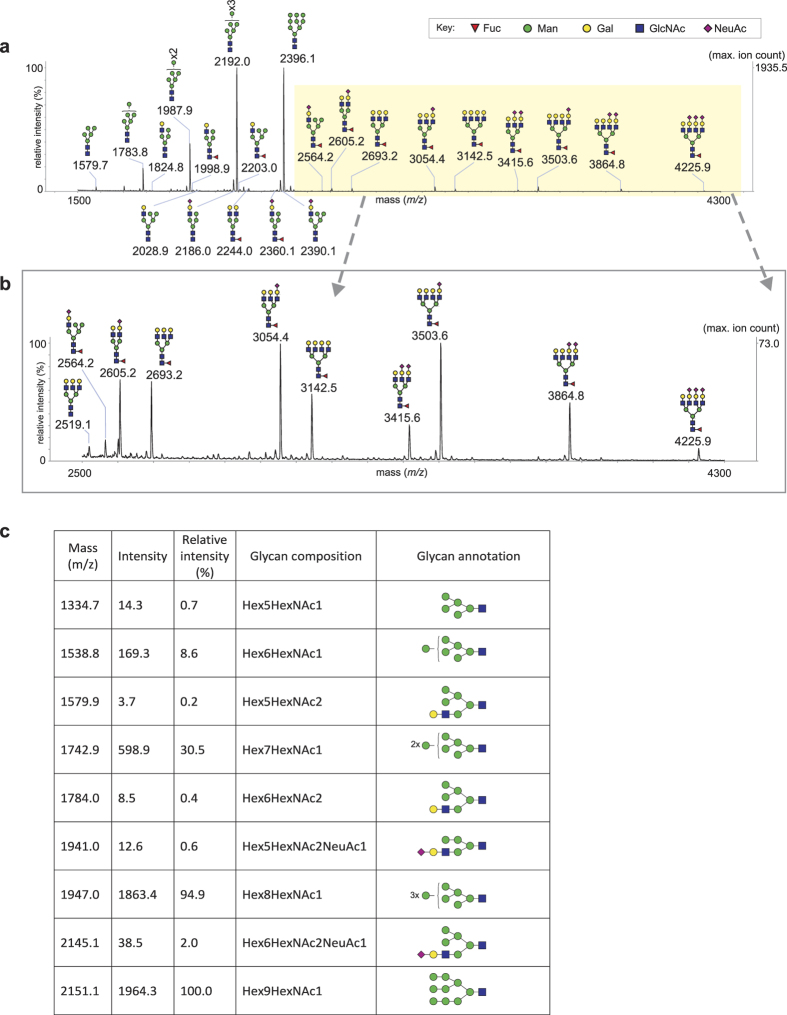
N-glycome of HIV-1 BaL gp120. MALDI TOF mass spectrum of N-glycans released after PNGase-F digest: **(a)** full range spectrum (m/z 1500–4300); **(b)** expanded region (m/z 2500–4300), highlighted in the full range mass spectrum. Signal abundance (relative intensity) is normalised to the most abundant ion (indicated on the right hand axis) of the specified mass range. Structural assignments were based on MS and MS/MS data and knowledge of N*-*glycan biosynthetic pathways. The satellite peaks near the major peaks are permethylation artefacts whilst the peaks which are labelled with an “x” are derived from known contaminants. **(c)** Tabulated MALDI TOF data for N-glycans released after Endo-H digest.

MALDI profiles of PNGase F and A digests were dominated by oligomannose glycans, with the most abundant having six to nine mannoses (Fig. 4a,b and Supplementary Fig. S1). Bi-, tri- and tetra-antennary complex type glycans were also observed. They were estimated to contribute about 10–15% of the N-glycome. The complex type glycans were mainly core fucosylated and their antennae were either uncapped or sialylated with a maximum of one, two or three sialic acids on the bi-, tri- and tetra-antennary structures, respectively. Glycoproteomic experiments (see later) suggested that hybrid glycans constitute a portion of the glycans at some sites. This was supported by a series of minor ions in the Endo H-derived N-glycome whose compositions were consistent with hybrid structures carrying non-capped or sialylated complex-type antennae (Fig. 4c).

### Glycoproteomics of gp120_BaL_ defines site occupancy

Table 1 and Supplementary Tables S1–S15 document the large body of data acquired from the numerous glycoproteomic analyses that enabled us to determine the types of glycans present at all consensus sites in gp120_BaL_. Below we present a concise summary of our structural conclusions for each glycosite. For clarity and convenience we have grouped the sites according to the domains of gp120 in which they are found.

#### The C1 domain

This domain has a single site (N1) at Asn-87. MALDI and ES analysis of tryptic and Endo H digests showed that this site has exclusively oligomannose glycans (Man_6–9_GlcNAc_2_) with Man_8_ being the most abundant.

#### The V1 and V2 domains

Seven glycosites are found in the V1/V2 domains. Three of these (N2, N6 and N8) are contiguous to the two S-S bridges that underpin the V1/V2 loops. N4, N5, and N6 are clustered in the N-terminal half of the V1 loop and N7 is three residues away from the S-S bridge at the beginning of the V2 loop.

The dominant glycans at N2 (Asn-129) are the same as at N1 (Man_6–9_ GlcNAc_2_). However, unlike N1, this site has a tiny amount of non-sialylated complex-type glycans (estimated to be about 0.3%). Their compositions (Hex_6–7_HexNAc_5–6_Fuc_0–1_) correspond to tri- and tetra-antennary glycans.

N3 (Asn-135) was the only site found to be exclusively glycosylated with complex-type glycans. All were sialylated with compositions of NeuAc_1_Hex_6_HexNAc_5_Fuc_1_, NeuAc_1_Hex_7_HexNAc_6_Fuc_1_ and NeuAc_2_Hex_7_HexNAc_6_Fuc_1_, corresponding to tri- and tetra-antennary core fucosylated structures. The disialylated tetra-antennary glycan was the most abundant.

Sites N4 (Asn-140) and N5 (Asn-143) are located three residues apart within a single tryptic glycopeptide (NVTNTTSSSR) and could not be studied separately. Acquiring glycoproteomic data on the intact glycopeptide was challenging, probably due to its low hydrophobicity, and only weak data for oligomannose glycoforms were obtained. Analysing Endo H digests by ES-MS/MS was more successful, however. Data were observed for three glycoforms of the truncated glycopeptide substituted with either two HexNAc residues, two HexNAcFuc moieties or one of each. Because Endo H cannot digest complex-type glycans, the HexNAc Fuc moieties must have originated from hybrid glycans. Therefore we conclude that N4 and N5 carry a mixture of oligomannose and hybrid glycans.

Like N4 and N5, sites N6 (Asn-159) and N-7 (Asn-163) are close to each other in the protein and were studied together. Their analysis was somewhat complicated by the presence of an I to K sequence variant at position 168 which resulted in two tryptic glycopeptides being present in the digests: NCSFNITTGIR and NCSFNITTGK. Nevertheless high quality data were obtained showing that these two sites are oligomannose of aggregate composition Man_14–18_GlcNAc_4_.

The N8 (Asn-200) tryptic glycopeptide gave only weak data despite numerous experiments. Therefore minor glycoforms will not have been observed. Molecular ions were observed consistent with sialylated hybrid and non-sialylated complex-type glycans. The presence of hybrid glycans was confirmed by Endo H digests. There was no evidence for oligomannose at this site.

#### The C2 domain

Sites N9, N10 and N11 are found in the C2 domain. N9 (Asn-244) and N10 (Asn-265) are located in a large tryptic glycopeptide spanning residues 240 to 276. Their glycans are exclusively oligomannose with an aggregate composition of Hex_12–19_GlcNAc_4_ with Man_8–9_GlcNAc_2_ being the most abundant glycans. The Hex_19_ composition is predicted to result from incomplete removal of glucose from the precursor glycan.

N11 was found to be a mixture of oligomannose and hybrid (estimated 8:1 relative abundance). This site has Man_5_GlcNAc_2_ in addition to the Man_6–9_GlcNAc_2_ compositions observed at the previous sites. Also Man_6–7_GlcNAc_2_ are more dominant than Man_8–9_GlcNAc_2_. The hybrid structures have compositions Hex_6_HexNAc_3_, NeuAcHex_5–6_HexNAc_3_ and NeuAcHex_6_HexNAc_3_Fuc.

#### The V3 “oligomannose shield” domain

N12 (Asn-292), N13 (Asn-298) and N14 (Asn-304) are located in a large tryptic glycopeptide spanning Cys-299, which, with Cys-333, constitutes the S-S bridge that defines the V3 loop. Their glycans are almost exclusively oligomannose with an aggregate composition of Man_22–26_GlcNAc_6_ with Man_24_GlcNAc_6_ dominating. An estimated up to 1% of the glycome of these three sites is hybrid. N15 (Asn-334) is on the opposite side of the V3 loop, next to the S-S bridge. Only oligomannose glycans were observed at this site: Man_5–9_GlcNAc_2_ with Man_8–9_GlcNAc_2_ dominating.

#### The C3 domain

There are two glycosylation sites in this domain. The first, N16 (Asn-341), carries oligomannose glycans (Man_6–9_GlcNAc_2_) only, whilst the second (N17; Asn-357) has a more diverse array of glycans. All three classes of glycans were observed at this site (oligomannose, complex and hybrid) and no class dominates. A complete list of glycan compositions is given in Table 1. The most abundant glycans observed were Man_7_GlcNAc_2_ (oligomannose), Hex_6_HexNAc_5_Fuc (non-sialylated complex) and NeuAcHex_5-6_HexNAc_3_Fuc (sialylated hybrid).

#### The V4 domain

This domain is very heavily glycosylated in the gp120 analysed here having 5 glycosites in the 28 amino acid (AA) loop formed by the S-S bridge between Cys-387 and Cys-415. The glycosites are N18 (Asn-387), N19 (Asn-393), N20 (Asn-397), N21 (Asn-403) and N22 (Asn-408). There are no tryptic cleavage sites in this domain and the large tryptic glycopeptide spanning His-364 to Arg-416 was not observed. Glycopeptide data were, nevertheless, successfully obtained on Endo H digests of chymotryptic and Glu-C sub-digests of the tryptic glycopeptide. HexNAc was observed at each site indicating that N18 to N22 are predominantly substituted with oligomannose glycans. The possibility that some of the glycans are hybrid cannot be ruled out, although the very low abundance of hybrid glycans in the Endo H glycomics data means that, if present, they are very minor.

#### The C-terminal domain

The polypeptide on the C-terminal side of the V4 loop is largely conserved in gp120s except for a small variable domain (V5) after the last S-S bridge. N23 (Asn-445) is located in a conserved part of the sequence three residues from this bridge. N24 (Asn-460) is 15 AA residues further down the sequence and is within the V5 domain. The former has exclusively oligomannose glycans (Man_5–9_GlcNAc_2_) with Man_8–9_GlcNAc_2_ being most abundant. In contrast, the latter (N24) is decorated with complex-type and hybrid glycans. Sialic acid is present on some of the hybrid glycans but not the complex glycans (Table 1).

## Discussion

The focus on HIV-1 Env as a vaccine target, and the development and detailed characterization of bnAbs directed against HIV-1gp120, including potent bnAbs that include glycans as part of their recognition sites, have increased interest in better understanding the glycan composition of this heavily glycosylated viral protein. However, until now, all of the experiments used to comprehensively characterise the site specific N-glycosylation of this glycoprotein have been based on analyses of recombinant expressed forms of gp120. Characterization of site-specific N glycosylation of gp120 derived from HIV-1 virions, the actual target of bnAbs, should allow a better understanding of what these bnAbs actually are recognising, and may facilitate the development of improved glycan-binding bnAbs and/or Env based vaccine immunogens.

In this study we determined the types of N-linked glycans that are present on each of the 24 N-glycan consensus sites of gp120 derived from virions produced by the HIV-1 BaL/SUPT1-R5 cell line [CLN204]. The characterisation of such a high number of glycosylation sites on a glycoprotein which could not be readily isolated in analysable quantities, was exceptionally challenging. We achieved this by employing an integrated glycomics and glycoproteomics strategy which takes advantage of the inherent specificity of three endo-glycosidases (PNGase F, PNGase A and Endo H), three proteases (trypsin, chymotrypsin and Endoproteinase Glu-C) and the application of both MALDI- and electrospray mass spectrometry.

In terms of overall glycan content, the virion derived gp120^SU^ we analysed is predominantly glycosylated with oligomannose structures which are mainly Man_9_GlcNAc_2_ (≈40%) and Man_8_GlcNAc_2_ (≈40%) with lesser amounts of Man_7_GlcNAc_2_ (≈15%), Man_6_GlcNAc_2_ (<5%) and Man_5_GlcNAc_2_ (<1%) (Fig. 4a). We also found significant quantities of complex-type glycans (10–15% of total glycome) together with minor amounts of hybrid N-glycans (<5% of total glycome) (Fig. 4a,b). The majority of the complex glycans are core fucosylated, sialylated and highly branched. Thus bi-, tri- and tetra-antennary glycans comprise about 15%, 40% and 45% of the complex-type glycome, respectively (Fig. 4b). The most abundant hybrid glycan is sialylated (Fig 4c). These results are broadly in line with previous glycomic investigations of viral derived gp120^8^,^17^,^22^ (reviewed in Doores *et al.*^18^). Scanlan and colleagues were the first to show marked differences between virion and recombinant gp120^8^,^17^. They found oligomannose contents of 56–79%, depending on the strain, which were substantially higher than the 30% expressed on recombinant material. This early work suggested that Man_5_GlcNAc_2_ was the most abundant oligomannose glycan in gp120_JRCSF_. However, a recent study by Pritchard *et al.* has shown that Man_7–9_GlcNAc_2_ compositions are actually the most abundant oligomannose components in this strain^22^. Our observations for the BaL strain are in accord with this result. Pritchard *et al.*^22^ employed ion mobility mass spectrometry to study the complex-type glycans in gp120_JRCSF_ and they found that the majority were sialylated multi-antennary structures. We have identified a similarly restricted repertoire in BaL. None of the aforementioned publications reported the presence of hybrid glycans on virion-derived gp120. Whether this is due to strain-specific expression of hybrid glycans, or to differing capabilities of the analytical methods used in the various studies, remains to be established.

**Figure 5:**
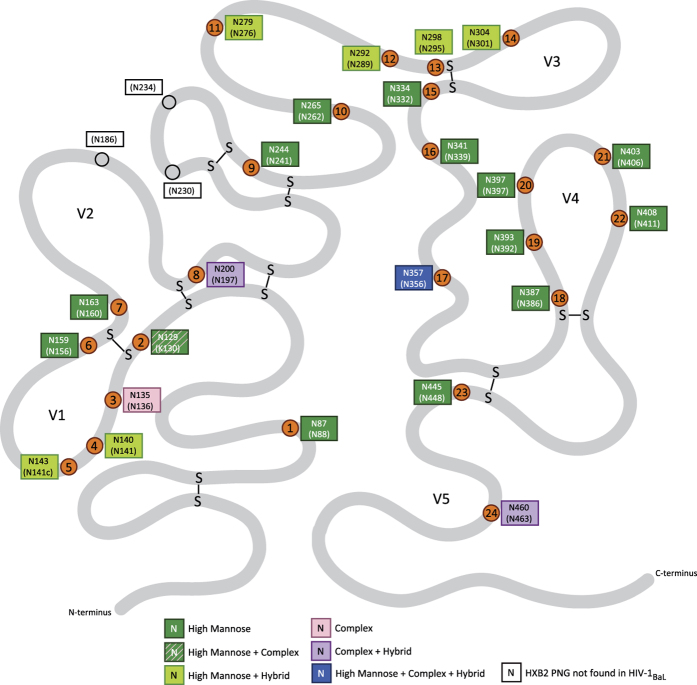
Visual representation of the HIV-1_BaL_ gp120 secondary structure. Residue numbers for N-linked glycosylation sites are listed as both sequential numbering with respect to the experimentally deduced BaL sequence, and with the numbering with respect to the reference HIV_HXB2_ indicated in brackets. Individual sites are indicated as orange circles, with the sequential site number (1–24) shown within the circle. The three consensus sites of HXB2CG not present in this swarm are indicated with empty circles. The general type of glycosylation (high mannose, complex, hybrid) observed at each site is indicated by colour coding, with the key given within the figure.

Our work constitutes the first systematic glycoproteomic analysis of any virion-derived gp120 to include site specific analysis. Other workers^23^ have previously applied glycoproteomic methods to viral gp120 but the focus of their work was the development of an automated spectral-aligning strategy and they only reported on five glycosylation sites. As shown in Fig 5, the virion-derived gp120 we studied has 24 N-glycosylation sites. Compared to the canonical HIV-1_HXB2_ strain, it is missing 3 N-glycosylation sites in and near the V2 loop and has an additional site in the V1 loop (Fig. 5). Thirteen sites are exclusively occupied with oligomannose glycans (Fig.5; dark green annotations). An additional seven sites are almost entirely oligomannose together with a trace (<0.3%) of non-sialylated complex structures (Asn-129) or low levels (<1%) of hybrid structures (Asn-140, Asn-143, Asn-279, Asn-292, Asn-298 and Asn-304). Complex-type glycans are found in substantial amounts at four sites. Two of these, Asn-200 and Asn-460, additionally carry hybrid structures, although oligomannose glycans are not present. A single site, Asn-357, carries oligomannose, complex and hybrid glycans. All three glycan classes are present at this site in comparable amounts. Finally, one site, Asn-135, is exclusively occupied with sialylated multiantennary complex glycans.

Much of current research aimed at understanding how broadly neutralising antibodies can penetrate the glycan shield of the HIV-1 envelope exploits recombinant Env trimers^24^,^25^. Although no trimer glycoproteome has yet been comprehensively mapped, information is available for about 75% of the glycosites in the soluble trimer BG505 SOSIP.664 expressed by HEK293T cells^11^,^12^. Thus, Behrens *et al.* quantified glycan populations at 17 of the 24 N-glycosites in the gp120 portion of this trimer. Ten sites were found to be exclusively occupied with oligomannose (Asn-156, Asn-234, Asn-262, Asn-295, Asn-332, Asn-339, Asn-363, Asn-386, Asn-392 and Asn-448; numbering corresponds to BG505 SOSIP.664), one site was mostly oligomannose with low levels of hybrid and complex (Asn-276), one site was occupied with both oligomannose and complex (Asn-160), three sites were mostly complex with low levels of oligomannose and hybrid (Asn-88, Asn-190 and Asn-462), and two sites were occupied with substantial quantities of all three classes of glycan (Asn-197, Asn-355). The main conclusions that can be drawn from comparison of the glycoproteomics data from the virion BaL and recombinant BG505 trimer are (i) the unprocessed oligomannose “patch” centred on the V3 domain is shared by both samples, and (ii) the recombinant trimer has substantially higher levels of complex glycans than virion BaL. Thus, Asn-88 is occupied mainly by complex glycans in the recombinant trimer whilst it is exclusively oligomannose in virion BaL (Asn-87); Asn-160 carries about 30% complex glycans in the recombinant BG505 trimer but none in BaL (Asn-163); Asn-190, which is almost entirely occupied with complex glycans in the trimer, is not a glycosite in BaL. Unfortunately the site that is exclusively occupied with multi-antennary complex glycans in virion BaL (Asn-135) was not characterised in the recombinant trimer study (Asn-137), so no conclusions relating to antibody recognition of this site can be drawn from the glycoproteomic comparisons.

The N-glycan site specific characterisation of virion-derived gp120 will be of great value in allowing a better understanding of the actual binding partners of a range of important bnAbs (Reviewed in Doores *et al.*^18^). The unusual domain exchanged 2G12 bnAb has been demonstrated to recognize Manα1,2Man-linked sugars of oligomannose glycans (Man_8–9_GlcNAc_2_) associated with sites N-295, N-332, N-339 and N-392 of the so called high-mannose patch of V3^26^,^27^. This is fully consistent with our present glycoproteomics data, which show that all these sites (N-298, N-334, N-341 and N-393) express Man_8-9_GlcNAc_2_ N-glycans. Additional bnAb such as PGT121–123 also bind the high mannose patch and N-332 as well as overlapping epitopes. The binding of PGT121–123 can include the Man_8–9_GlcNAc_2_ N-glycan at N-332 and associated glycans in the V1/V2 loops such as N-136 and N-156. Binding of PGT121–123 to viruses produced in N-acetylglucosaminyltransferase 1 (GnT1)^−/−^ cells, which only express oligomannose N-glycans, indicates little or no requirement for complex type glycans. However, if the N-332 glycan is absent a requirement for complex glycans in V1/V2 sites such as N-136 and N-156 was deemed to be important^28^. As indicated above, our data show that N-334 does express Man_8–9_GlcNAc_2_ N-glycans. Also consistent with these observations is that N-135 (equivalent to N-136) expresses exclusively complex type N-glycans. However, N-159 (equivalent to N-156) expresses oligomannose N-glycans and no complex glycans were detected at this site.

A family of bnAb, including PG9 and PG16, have been described that bind the trimer apex of the V1/V2 area which include the N-160 glycosylation site. X-ray crystallographic structural studies of protein scaffolds of the V1/V2 region with bound PG9 demonstrated the critical importance of a Man_5_GlcNAc_2_ N-glycan structure at N-160. Additional interactions with the protein bound GlcNAc of the N-glycans at N-156/173 were also indicated to be important^29^. Subsequent structural analysis of PG9 and PG16 interacting with protein scaffolds of the V1/V2 region also demonstrated the importance of oligomannose type glycans (at least Man_5_GlcNAc_2_) at N-160 but showed binding of a sialylated complex or hybrid N-glycan at N-156/173^30^. Our data show the equivalent site of N-160 on the gp120 we studied, N-163, is substituted with oligomannose glycans. In this gp120 the equivalent site to N-173 does not contain an N-glycosylation site and the equivalent site to N-156 (N-159) contains oligomannose glycans. There was no evidence for complex N-glycans at either N-159 or N-163 in BaL.

An additional set of bnAb, for example VRCO1, recognize the CD4 binding site on gp120. X-ray crystallography studies with recombinant gp120 and VRCO1 have demonstrated interactions with the protein bound GlcNAc of the N-glycans at N-276. To facilitate crystallization of the gp120, Endo H digestion was used which left only a GlcNAc or possibly GlcNAcFuc residues, which indicate that the N-glycans at site N-276 must be either oligomannose or hybrid glycans^31^. This is fully consistent with our current data which indicate that the equivalent glycosylation site N-279 expresses both oligomannose and core fucosylated hybrid N-glycan structures.

In conclusion, we have achieved the first systematic glycosylation site analysis of a gp120 derived from virions produced by infected T lymphoid cells. This is an important achievement because the strategies we have optimised constitute the blueprint for future comparative characterization of a variety of virion derived envelope glycoproteins of different virus isolates from different cell sources. Such analyses should provide insights into optimizing the design of immunogens as well as methods and reagents for evaluation of neutralizing antibodies, including those generated in response to vaccination.

## Methods

### Derivation of the HIV-1 BAL/SUPT1-CCR5 CL.30 cell line

All work was performed using aseptic techniques in a biosafety level 2 laboratory following biosafety level 3 practices. The cell line, HIV-1 BAL/SUPT1-CCR5 CL.30, designated cell line number 204 (CLN204), was derived by infecting the SUPT1-CCR5 CL.30 cell line CLN195 which is a limiting dilution clone of the SUPT1-CCR5 cell line previously described^32^ with an infectious virus stock of HIV-1 BAL/Human peripheral blood lymphocytes (PBL) obtained from the NIH AIDS Reagent Program (cat.# 510, lot # 97116). The HIV-1 BAL/SUPT1-CCR5 CL.30 cell line is a bulk HIV-1 infected cell line. SUPT1-CCR5 CL.30 cells were propagated using RPMI1640 supplemented with 10% Fetal Bovine Serum (heat-inactivated), 100 units/mL Penicillin and 100 μg/mL Streptomycin (complete medium) in T flasks and passaged 1:3 twice weekly. CCR5 transgene expression by SUPT1-CCR5 CL.30 cells is under Puromycin selection (0.3 μg/mL), although Puromycin selection was not required to maintain CCR5 expression. The cell density was typically ~1.0 × 10^6^ viable cells/mL and >90% viability at each passage. The fluorescence-activated cell sorting (FACS) immunophenotype of these cells was found to be essentially 100% CD4 and CXCR4 positive with an average of 67% CCR5 positive. Cells were 100% human leukocyte antigen (HLA) Class I positive and HLA Class II negative.

For infection, a cell pellet consisting of 1.5 × 10^7^ viable SUPT1-CCR5 CL.30 cells was prepared by centrifugation at ~600 × g in a 15 mL polypropylene tube and inoculated with 0.5 mL of the HIV-1 BaL infectious stock. An equal volume of complete media containing 4 μg/mL polybrene was added to achieve a final concentration of 2 μg/mL polybrene and the cells incubated at 37 °C for 2 hours. The cells were transferred to a T75 flask containing 30 mL of complete media containing 2 μg/mL polybrene and the culture incubated at 37 °C in 5% CO_2_ overnight. Polybrene and unbound virus were removed from the cells by centrifugation as described above. The cell pellet was transferred to a new T75 flask containing 30 mL of complete media and returned to the incubator. This culture was passaged twice weekly and monitored for progeny virus production using an in-house HIV-1 p24^CA^ antigen capture immunoassay. By day 4 post infection (PI), virus induced cytopathic effects (CPE) were evident and both the cell density and viability were declining. To maintain culture viability and increase virus yield, ~500,000 non-infected SUPT1-CCR5 CL.30 cells were added per mL of culture. At the next three passages, non-infected cells were also required and added after which the culture was allowed to undergo crisis during which the culture viability dropped to 2%. By day 42 PI a stable, productively infected, CPE-free cell line arose that when passaged twice weekly at 1:3 yielded ~1.0 × 10^6^ viable cells/mL and >90% viability. This cell line has been cultured for over 85 passages in T flasks and consistently provided 1–2 × 10^3 ^ng/mL HIV-1 p24^CA^ as assessed by antigen capture immunoassay, ~1.0 × 10^6^ viable cells/mL and >90% viability at the day of passage. The flow cytometric immunophenotype of this cell line is CD4 negative, CCR5 negative, 100% CXCR4 positive, and typically >85% intracellular HIV-1 gag positive. It is also 100% HLA Class I positive and HLA Class II negative.

### Viral tropism assay

HIV-1 co-receptor usage was determined by inoculating a trio of CD4 expressing cell lines derived from SUPT1 cells which express either: no co-receptor, the CXCR4 co-receptor or the CCR5 co-receptor^33^. Cell cultures were inoculated with non-concentrated virus stocks and monitored for virus infection by observing for cytopathic effects and by HIV-1 p24^CA^ antigen capture immunoassay for up to three weeks. Substantial cytopathic effects and/or increasing HIV-1 p24^CA^ concentrations were used to detect infection and thus determine co-receptor usage for the virus tested.

### TZM-bl Assay

The TZM-bl cell line, initially referred to as JC53BL cells, were obtained from the NIH AIDS Reagent Program (Cat.# 8129) and propagated as monolayers in T-flasks essentially as previously described. Infection of this cell line with HIV-1 results in the trans-activating transcriptional activator (TAT) driven expression of β-galactosidase and luciferase reporter cassettes. Infectious titres of HIV-1 in culture supernatant samples were determined using 96 well plates seeded with 1.0 × 10^4^ viable TZM-bl cells per well in 100 μl of phenol red free-RPMI1640 media containing 10% foetal bovine serum (heat inactivated), 2 mM L-glutamine, and 100 U/mL penicillin and 100 μg/mL streptomycin (=assay media). Plates seeded with these cells were incubated at 37 °C in 5% CO_2_ for 3 to 18 hours to allow the cells to attach. Serial, 3-fold dilutions of samples were prepared in assay media. After aspirating the culture supernatant, duplicate (100 μl) samples of each dilution were added and the plate(s) incubated for 72 hours at 37 °C in 5% CO_2_. The presence of replicating HIV-1 was detected via the β-galactosidase reporter cassette. After aspirating the culture supernatant, 100 μl of a solution containing 1% Triton X-100, 1.5 mM Chlorophenol Red-β-Galactopyranoside (Roche cat.# 10884308001), and 0.12% NuPAGE reducing agent (Life Technologies cat.# NP0004) in DPBS containing calcium and magnesium was added to each well. Colour development was allowed to proceed for 1 hour while incubating at 37 °C and the absorbance of each well determined using a microplate reader (Molecular Devices VMax^®^). Reciprocal titres were determined by interpolation between the average absorbance of the two reciprocal dilutions that bracketed the arbitrary 1.0 absorbance unit cut-off. An aliquot of a positive control HIV-1 infectious stock sample (stored at −80 °C) was rapidly thawed in a 37 °C water bath and its titre determined in each assay to monitor for assay reproducibility.

### Determination of infectious titre

Serial, ten-fold dilutions of culture supernatant samples containing HIV-1 were prepared in complete media and a 1 mL portion of each dilution added to a 5.0 × 10^6^ cell pellet of SUPT1-CCR5 CL.30 cells in a sterile 15 mL polypropylene tube. The cell pellets were gently resuspended in their respective samples and, after incubation at 37 °C for an hour, transferred to T25 flasks containing 9 mL of complete media. Cultures were incubated at 37 °C in 5% CO_2_ and split 1:2, twice weekly. The concentration of HIV-1 p24 in them was determined by HIV-1 p24 antigen capture immunoassay every 7 days for 21 days. Sample infectious titre was defined as the greatest reciprocal dilution resulting in a positive HIV-1 p24 antigen capture immunoassay result.

### Large scale production and purification of virus

In brief, large scale culture propagation was performed using 850 cm^2^ roller bottles containing 400 mL of culture in complete media. Cultures were harvested and passaged 1:2 twice weekly. Cells were removed from harvested material by 5.0 μm capsule filtration (Millipore Polygard CN Opticap XL10, Cat. No. KN50A10HH1). The virus from up to 30 litre batches of culture filtrate was purified and concentrated by continuous flow-sucrose density gradient centrifugation in a 25 to 50% sucrose in Tris/NaCl/EDTA (TNE) gradient using a Beckman CF32 rotor at 30,000 rpm at 4 °C with a flow rate of less than 6 litres per hour. Virus containing sucrose density gradient fractions were diluted to less than 20% sucrose and the sucrose removed by direct pelleting the virus in a fixed angle Beckman TY45 rotor at 30,000 rpm for 1 hour at 4 °C. The virus pellet was resuspended in sterile TNE at a final concentration of 1000x (i.e. 1 litre of cell culture yielded 1 mL of purified virus) and 250 μl aliquots were stored in liquid N_2_ vapour. Typical lots contained ~2.6 mg/mL of total protein (Bio-Rad DC Assay) and between 0.3 and 0.5 mg/mL capsid protein. The purified virus lots P4235, P4236, P4238 and P4239 were used for the glycan experiments.

### Viral genetic analysis

**Figure 6:**
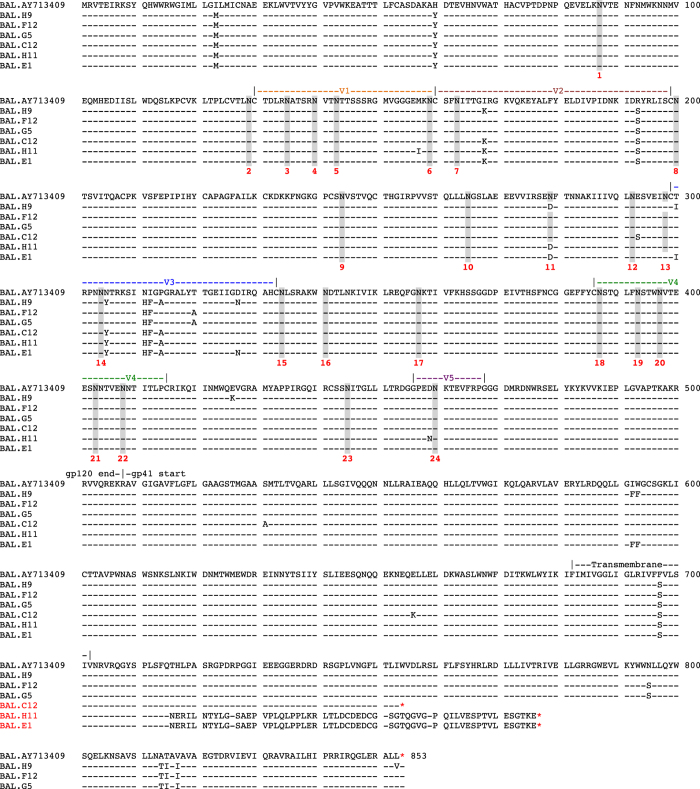
Complete gp160 amino acid alignment of HIV-1 BaL/SupT1-R5. BAL.AY713409 is the published reference sequence (GenBank accession number AY713409). Dashed lines show sequence identity to the reference sequence and amino acid polymorphisms are indicated by a single letter amino acid abbreviation. Key landmarks in gp160 are denoted above each region. Potential N-linked glycosylation sites are shaded. In two sequences (H11 and E1), a nucleotide frame shift lead to a premature stop codon (*). In a third sequence (C12) a nucleotide change in W754 also led to a premature stop codon (*).

Single genome amplification (SGA) was utilized to generate unique, independent sequences from cultured HIV-1 BaL/SUPT1-CCR5 cells, as previously described^34^. The entire 3′ half of the viral genome (including the entire vif, vpr, vpu, tat, rev env and nef genes) was amplified from DNA (Qiagen DNA Blood kits). PCR was performed using a limiting dilution approach where only one amplifiable molecule was present in each reaction. PCR was performed with 1 × PCR buffer, 2 mM MgCl_2_, 0.2 mM of each deoxynucleoside triphosphate, 0.2 μM of each primer, and 0.025 U/μL Platinum Taq polymerase (ThermoFisher) in a 20 μL reaction. First round PCR was performed with sense primer HIVBK3F1 5′-ACAGCAGTACAAATGGCAGTATT-3′ and antisense primer HIVR3B3.R1 under the following conditions: 1 cycle of 94 °C for 2 min, 35 cycles at 94 °C for 15 sec, 55 °C for 30 sec, and 72 °C for 4 min, followed by a final extension of 72 °C for 10 min. Next, 1 μL from the first-round PCR product was added to a second-round PCR reaction that included the sense primer HIVBK3F2 5′-TGGAAAGGTGAAGGGGCAGTAGTAATAC-3′ and antisense primer HIVR3B6.R2 5′-TGAAGCACTCAAGGCAAGCTTTATTGAGGC-3′ performed under the same conditions used for first-round PCR, but with a total of 45 cycles. Correct sized amplicons were identified by agarose gel electrophoresis and directly sequenced with second round PCR primers and HIV specific primers using BigDye Terminator technology. Sequences were aligned using ClustalW and hand edited using MacClade 4.08. The entire gp160 amino acid alignment is shown in Fig. 6.

### Sypro Dual colour fluorescent protein gel analysis

Proteins from lysed virus preparations were resolved by SDS-PAGE on 4–20% Tris-glycine gels (Invitrogen) under reducing conditions. The p24 and gp120 content of the samples were determined by a two-colour fluorescence staining assay. Gels with virus samples and a dilution series of purified protein standards were stained with two fluorescent dyes (Molecular Probes, Eugene, OR), SYPRO Pro-Q Emerald (green fluorescence) to detect glycoproteins, such as Env, and SYPRO Ruby (red fluorescence) to detect all proteins, including p24. Stained gels were analysed for fluorescence at 520 nm with UV excitation, using a VersaDoc 3000 Imaging System (Bio-Rad Laboratories, Hercules CA) software package (Bio-Rad Laboratories) by interpolating the integrated pixel density signals from the unknown samples onto a standard curve derived from a linear regression of density values for serial dilutions of highly purified, quantitative amino acid analysis quantified standards, either recombinant vaccinia-produced HIV-1_MN_ gp120^SU^ (generously provided by Drs. B. Puffer and R. Doms, University of Pennsylvania, Philadelphia, PA) or HIV-1_MN_ virion derived p24^CA^ (AIDS and Cancer Virus Program, Frederick National Laboratory, Frederick, MD). Well characterized reference preparations of SIVmac239/SUPT1-R5 and HIV-1_MN_ /H9 Clone 4 (AIDS and Cancer Virus Program, Frederick National Laboratory, Frederick, MD) were used to validate this procedure^15^,^35^.

### Reverse-phase high performance liquid chromatography (RP-HPLC) purification of viral gp120 proteins

Viral samples were disrupted in 8 M Guanidine-HCl (Pierce, Rockford, IL) and fractionated under non-reducing conditions by HPLC to isolate viral gp120 protein. HPLC was performed at a flow rate of 300 μL/min on 2.1 × 100 mm Poros^®^ R2/H narrow bore column (Boehringer Mannheim GmbH, Germany), using aqueous acetonitrile/trifluoroacetic/acid solvents and a Shimadzu HPLC system equipped with LC-10AD pumps, SCL-10A system controller, CTO-10AC oven, FRC-10A fraction collector and SPD-M10AV diodearray detector. The gradient of buffer B (0.1% trifluoroacetic acid in acetonitrile) was: 10–36.5%, 12 min; 36.5–37%, 4 min; 37–41%, 7 min; 41–70%, 12 min; and 70%, 5 min. A temperature of 55 °C was maintained during HPLC separation. Peaks were detected by UV absorption at 206 nm and 280 nm. Fractions containing HIV-1 BAL/SUP-T1-R5 gp120 were lyophilized for further SDS-PAGE purification.

### SDS-PAGE purification of viral gp120 protein

In adjacent gel lanes, gp120 purified from the HIV-1 BAL/SUPT1-R5 viral samples by HPLC and the viral samples disrupted in 2x sample buffer were loaded on a 1.5 mm thick 4–20% Tris glycine gel (Invitrogen, Carlsbad, CA). The samples were separated by SDS-PAGE, followed by staining with Coomassie R-250. After destaining the gp120–containing bands were excised and transferred in tubes with 1% acetic acid solution.

### Production of gp120 tryptic glycopeptides by in-gel digestion

Excised bands corresponding to gp120 were first destained in a 50% acetonitrile (MeCN) (v/v) solution in 0.1 M ammonium bicarbonate for 10 min, followed by incubation with 10 mM dithiothreitol (DTT) solution for 30 min at 56 °C. The gel pieces were then desiccated prior to incubation with a 55 mM solution of iodoacetic acid for 30 min at room temperature in the dark. The gel pieces were desiccated again prior to incubation with 1 μg of porcine trypsin (EC: 3.4.21.4, Promega) in 50 mM ammonium bicarbonate, pH 8.5 (adjusted with ammonia), overnight at 37 °C. Following extraction of the tryptic glycopeptides from the gel in acetonitrile/0.1% trifluoroacetic acid (6:4 v/v), the eluent volumes were reduced to approximately 10 μL under vacuum^36^,^37^. The overall methodological approach used is summarised in Fig. 3.

### Glycomic analyses

For glycomic experiments, the tryptic peptide/glycopeptide digest mixture was first subjected to enzymatic release of the N-glycan populations, before being purified, derivatised and analysed by MALDI-TOF-MS and MS/MS. The overall glycomic analytical approach is summarised in the upper half of Fig. 3 38,39,40.

#### N-linked glycan release by PNGase F

Aliquots of the reduced, carboxymethylated and trypsin-digested gel extracts were diluted by the addition of 200 μL fresh 50 mM ammonium hydrogen carbonate, pH 8.4 (adjusted with ammonia). 0.5U of N-glycosidase F (PNGase F) in glycerol (Roche EC 3.5.1.52) were then added and the sample incubated at 37 °C overnight before being terminated by lyophilisation.

#### N-linked glycan release by PNGase A

Aliquots of the reduced, carboxymethylated and trypsin-digested gel extracts were diluted by the addition of 200 μL fresh 50 mM ammonium acetate, pH 5.0 (adjusted with acetic acid). 0.5U of N-glycosidase A (PNGase A) in glycerol (Roche EC 3.5.1.52) were then added and the sample incubated at 37 °C overnight before being terminated by lyophilisation.

#### N-linked glycan release by Endo H

Aliquots of the reduced, carboxymethylated and trypsin-digested gel extracts were diluted by the addition of 200 μL 50 mM ammonium hydrogen carbonate, pH 8.4 (adjusted with ammonia). 0.5U of Endoglycosidase H (Endo H) (Roche EC 3.2.1.96) were then added, together with 5 μL of supplied Endo H Reaction Buffer. The reaction was then incubated at 37 °C overnight before being terminated by lyophilisation.

#### Separation of released glycans from peptides

Released glycans were separated from residual peptides using a reverse-phase C18 Sep-Pak cartridge. The Sep-Pak was conditioned successively with methanol, 5% acetic acid (aq., v/v), propan-1-ol, and 5% acetic acid. The sample was dissolved in 5% acetic acid, loaded onto the cartridge, and eluted successively with 5% acetic acid followed by 20%, 40%, 60% and 100% propan-1-ol in 5% acetic acid (v/v). The organic solvent was removed on a Savant Speed-Vac concentrator (ThermoFisher Scientific Inc.) and samples were lyophilized prior to permethylation.

#### Permethylation of released glycan pools

Permethylation was performed using the sodium hydroxide procedure^41^. Briefly, sodium hydroxide pellets were crushed with dimethyl sulfoxide (DMSO) to form a slurry. A 1 mL aliquot of this slurry was added to the dried glycans, followed by the addition of 1 mL of methyl iodide (ICH_3_). The mixture was vigorously mixed on an automatic shaker for 10 min at room temperature^42^. The reaction was terminated by the addition of 1 mL of water, and permethylated glycans were recovered by chloroform extraction. The chloroform layer was washed several times with water in order to remove impurities and was then dried under a stream of nitrogen. Permethylated N-glycans were purified using a reverse-phase C18 Sep-Pak cartridge. The Sep-Pak was conditioned successively with methanol, water, acetonitrile (MeCN), and water. The sample was dissolved in 1:1 (v/v) methanol-water, loaded onto the cartridge, washed with water and 15% (v/v) aqueous MeCN solution, and then eluted using a 75% (v/v) aqueous MeCN solution. The organic solvent was removed on a Savant Speed-Vac concentrator and samples were lyophilized prior to MALDI-TOF-TOF-MS and MS/MS analyses.

#### MALDI-TOF mass spectrometry of permethylated glycans

MALDI-TOF-MS data on permethylated samples were acquired in the reflector positive-ion mode using a 4800 MALDI-TOF/TOF (Applied Biosystems, Foster City, CA) mass spectrometer. The instrument was calibrated externally using the Calmix 4700 calibration standard, containing des-Arg1-bradykinin, angiotensin I, human [Glu1]-fibrinopeptide B, adrenocorticotropin (ACTH) fragment 1–17, ACTH fragment 18–39 and ACTH fragment 7–38. Samples were dissolved in 20 μL of methanol, and 1 μL was mixed at a 1:1 ratio (v/v) with 2,5-dihydrobenzoic acid (20 mg/mL in 50% (v/v) methanol in water) as a matrix. Then samples were spotted onto a 384-well sample plate and were dried under a vacuum. Data were acquired using 4000 Series Explorer instrument control software and were processed using Data Explorer MS processing software. MS spectra were assigned and annotated with the help of the GlycoWorkbench software^43^,^44^.

### Glycoproteomic analyses

For glycoproteomic experiments the tryptic peptide/glycopeptide gp120 digest mixture (see earlier) was analysed directly by on-line nano-LC-MS and MS/MS by electrospray ionisation (see *Nano-LC ES-MS and MS/MS analysis of glycopeptides*) and by MALDI via auto-spotted plates, or first subjected to N-linked glycan release by Endo H or PNGase F and/or sub digestion with additional proteases prior to analysis. The overall Glycoproteomic approach is summarised in the lower half of Fig. 3 36,45,46,47,48,49. Methods used for the proteolytic sub-digestions are presented below.

#### Glu-C sub digestion

Aliquots of the reduced, carboxymethylated, trypsin-digested gel extracts, following treatment by Endo H were digested with 1 μg of Endoproteinase Glu-C from Staphylococcus aureus strain V8 (EC:3.4.21.19) in 20 μL of 50 mM Tris (pH 8.0). The reaction was then incubated at 37 °C overnight before being terminated by lyophilisation.

#### Chymotrypsin sub digestion

Aliquots of the reduced, carboxymethylated, trypsin-digested gel extracts, following treatment by Endo H were digested with 1 μg of chymotrypsin from bovine pancreas (EC:3.4.21.4, Sigma) in 20 μL of 50 mM ammonium hydrogen carbonate (pH 8.4). The reaction was then incubated at 37 °C overnight before being terminated by lyophilisation.

#### Offline nano-LC-MALDI-TOF mass spectrometry of glycopeptides

Glycopeptide pools were dissolved in 40 μL 0.1% (v/v) trifluoroacetic acid (TFA) and separated by nano-LC using an Ultimate 3000 (Thermo Scientific Dionex (UK) Ltd, Camberley) fitted with a Pepmap analytical C18 nanocapillary column. After loading in 2% (v/v) acetonitrile in 0.1% TFA (v/v), the column was eluted with a gradient of acetonitrile in 0.1% TFA at a flow rate of 0.3 μL/min. Sample elutions were spotted directly onto a steel MALDI target plate using a Probot system (Thermo Scientific Dionex (UK) Ltd, Camberley) with α-cyano-4-hydroxycinnamic acid matrix at a concentration of 3.3 mg/mL. Peptides were subjected to MALDI-MS profiling, complemented with MS/MS sequencing of the 10 most abundant ions in each sample, on an Applied Biosystems 4800 MALDI-TOF/TOF mass spectrometer (Applied Biosystems, Foster City, CA) operated in reflector positive-ion mode. The instrument was calibrated externally using the Calmix 4700 calibration standard. Data were acquired using 4000 Series Explorer instrument control software and were processed using Data Explorer MS processing software.

#### Nano-LC ES-MS and MS/MS analysis of glycopeptides

The gp120 tryptic digest mixture, or collected glycopeptide pools from initial runs resuspended in 0.1% (v/v) TFA, were analysed by nano-LC-ES-MS/MS using a nano-high-performance liquid chromatography (HPLC) system (Thermo Scientific Dionex (UK) Ltd, Camberley) connected to a quadrupole TOF^49^ mass spectrometer (API Q-STAR^®^ Pulsar i, Applied Biosystems/MDS Sciex, Toronto, Canada). Separations were achieved by means of a 75 μm C18 reverse-phase column eluted with a gradient of acetonitrile in 0.01% formic acid at a flow rate of 200 nL/min. Data-dependent acquisition of MS/MS spectra was controlled by setting threshold ionization values for doubly, triply, and quadruply charged ions, and collision energies were set to produce good fragmentation. The instrument was pre-calibrated using 10–100 fmol/μL of [Glu1]-fibrinopeptide B /5% (v/v) acetic acid (1:3, v/v). In the MS/MS mode, the collision gas utilized was nitrogen and the pressure was maintained at 5.3 × 10^−5^ Torr. Data acquisition was performed using Analyst QS (Applied Biosystems, Darmstadt, Germany) software with an automatic information-dependent-acquisition function.

#### Interpretation of glycoproteomic data

This was done manually as previously described^36^,^45^,^46^,^47^,^48^,^49^ and is exemplified by the representative data shown in Supplementary Figures S2–S6. The spectra shown in this figure illustrate the good quality of the MS/MS data, despite the low ion counts of many of the glycopeptide molecular ions, an inevitable consequence of the limited amounts of sample available. Key elements of the logic associated with manual interpretation of MS/MS data is contained in the legend to Supplementary Figures S2–S6.

## Additional Information

**How to cite this article**: Panico, M. *et al.* Mapping the complete glycoproteome of virion-derived HIV-1 gp120 provides insights into broadly neutralizing antibody binding. *Sci. Rep.* **6**, 32956; doi: 10.1038/srep32956 (2016).

## Supplementary Material

Supplementary Information

Supplementary Tables S1-S15

Supplementary Table S16

## Figures and Tables

**Table 1:** Summary of observed site-specific glycosylation of HIV-1 BaL gp120.

Site	Sequential BaL numbering	Relative to HXB2CG	Tryptic Peptide	Observed Glycosylation			Glycoproteomics				Glycomics			Supplementary Table
				High Mannose	Hybrid	Complex	RCM-T	RCM-T + Endo-H	RCM-T + C + Endo-H	RCM-T + G + Endo-H	RCM-T + PNGase-F	RCM-T + PNGase-A	RCM-T + Endo-H	
N1	N87	N88	_87_NVTENFNMWK_96_	Man 6-9			✓	✓	✓		✓	✓	✓	S1
N2	N129	N130	_121_LTPLCVTLNC TDLR_134_	Man 6-9		Hex7 HexNAc6 Fuc								
						Hex7 HexNAc6								
						Hex6 HexNAc5 Fuc	✓	✓	✓		✓	✓	✓	S2
						Hex6 HexNAc5								
N3	N135	N136	_135_NATSR_139_			Hex7 HexNAc6 NeuAc2 Fuc								
						Hex7 HexNAc6 NeuAc Fuc	✓	✓			✓	✓		S3
						Hex6 HexNAc5 NeuAc Fuc								
N4	N140	N141	_140_NVTNTTSSSR_149_	Man 4-9				✓			✓	✓	✓	S4
N5	N143	N141c	_140_NVTNTTSSSR_149_	Man 4-9				✓			✓	✓	✓	S4
N6	N159	N156	_159_NCSFNITTGI R_169_	Man 6-9			✓	✓			✓	✓	✓	S5
N7	N163	N160	_159_NCSFNITTGI R_169_	Man 6-9			✓	✓			✓	✓	✓	S5
N8	N200	N197	_196_LISCNTSVIT QACPK_210_		Hex6 HexNAc3 NeuAc	Hex5 HexNAc4 Fuc	✓	✓	✓		✓	✓	✓	S6
					Hex5 HexNAc3 NeuAc Fuc	Hex6 HexNAc5 Fuc								
					Hex5 HexNAc3 NeuAc	Hex6 HexNAc5 Fuc2								
N9	N244	N241	_240_GPCSNVSTVQ CTHGIRPVVS_259 260_TQLLLNGSLA EEEVVIR_276_	Man 6-9			✓	✓	✓		✓	✓	✓	S7
N10	N262	N262	_240_GPCSNVSTVQ CTHGIRPVVS_259 260_TQLLLNGSLA EEEVVIR_276_	Man 6-9			✓	✓	✓		✓	✓	✓	S7
N11	N279	N276	_277_SENFTNNAK_285_	Man 5-9	Hex6 HexNAc3 NeuAc Fuc		✓	✓			✓	✓	✓	S8
					Hex6 HexNAc3 NeuAc									
					Hex5 HexNAc3 NeuAc									
					Hex6.HexNAc3									
N12	N292	N289	_286_IIIVQLNESV EINCTRPNNN_305 306_TR_307_	Man 6-9	Hex5 HexNAc3		✓	✓		✓	✓	✓	✓	S9
					Hex6 HexNAc3									
					Hex5 HexNAc3 NeuAc									
					Hex6 HexNAc3 NeuAc									
N13	N298	N295	_286_IIIVQLNESV EINCTRPNNN_305 306_TR_307_	Man 6-9	Hex5 HexNAc3									
					Hex6 HexNAc3									
					Hex5 HexNAc3 NeuAc		✓	✓		✓	✓	✓	✓	S9
					Hex6 HexNAc3 NeuAc									
N14	N304	N301	_286_IIIVQLNESV EINCTRPNNN_305 306_TR_307_	Man 6-9	Hex5 HexNAc3									
					Hex6 HexNAc3									
					Hex5 HexNAc3 NeuAc		✓	✓		✓	✓	✓	✓	S9
					Hex6 HexNAc3 NeuAc									
N15	N334	N332	_330_QAHCNLSR_337_	Man 5-9			✓	✓			✓	✓	✓	S10
N16	N341	N339	_340_WNDTLNK_346_	Man 6-9			✓	✓		✓	✓	✓	✓	S11
N17	N357	N357	_353_EQFGNK_358_	Man 5-9	Hex6 HexNAc3 NeuAc Fuc	Hex8 HexNAc7 NeuAc Fuc								
					Hex6 HexNAc3 NeuAc	Hex8 HexNAc7 Fuc								
					Hex5 HexNAc3 NeuAc Fuc	Hex7 HexNAc6 NeuAc Fuc								
					Hex6 HexNAc3 Fuc	Hex7 HexNAc6 Fuc								
					Hex5 HexNAc3 NeuAc	Hex7 HexNAc6								
					Hex6 HexNAc3	Hex6 HexNAc6 Fuc								
					Hex5 HexNAc3 Fuc	Hex6 HexNAc5 NeuAc Fuc								
					Hex5 HexNAc3	Hex6 HexNAc5 Fuc	✓	✓			✓	✓	✓	S12
						Hex5 HexNAc5 Fuc								
						Hex5 HexNAc4 Fuc								
						Hex5 HexNAc3 Fuc								
						Hex5 HexNAc4								
						Hex4 HexNAc4 Fuc								
						Hex4 HexNAc3 Fuc								
						Hex3 HexNAc3 Fuc								
N18	N387	N386	_364_HSSGGDPEIV THSFNCGGEF_383 384_FYCNSTQLFN STWNVTEESN_403 404_NTVENNTITL PCR_416_	Man 5-9			✓	✓	✓	✓	✓	✓	✓	S13
N19	N393	N392	_364_HSSGGDPEIV THSFNCGGEF_383 384_FYCNSTQLFN STWNVTEESN_403 404_NTVENNTITL PCR_416_	Man 5-9			✓	✓	✓	✓	✓	✓	✓	S13
N20	N397	N397	_364_HSSGGDPEIV THSFNCGGEF_383 384_FYCNSTQLFN STWNVTEESN_403 404_NTVENNTITL PCR_416_	Man 5-9			✓	✓	✓	✓	✓	✓	✓	S13
N21	N403	N406	_364_HSSGGDPEIV THSFNCGGEF_383 384_FYCNSTQLFN STWNVTEESN_403 404_NTVENNTITL PCR_416_	Man 5-9			✓	✓	✓	✓	✓	✓	✓	S13
N22	N408	N411	_364_HSSGGDPEIV THSFNCGGEF_383 384_FYCNSTQLFN STWNVTEESN_403 404_NTVENNTITL PCR_416_	Man 5-9			✓	✓	✓	✓	✓	✓	✓	S13
N23	N445	N448	_442_CSSNITGLLL TR_453_	Man 5-9			✓	✓			✓	✓	✓	S14
N24	N460	N463	_454_DGGPEDNKTE VFRPGGGDMR_473_		Hex6 HexNAc3 NeuAc Fuc	Hex7 HexNAc6 Fuc								
					Hex5 HexNAc3 NeuAc Fuc	Hex6 HexNAc5 Fuc								
					Hex6 HexNAc3 Fuc	Hex5 HexNAc5 Fuc								
						Hex5 HexNAc4 Fuc	✓	✓	✓		✓	✓	✓	S15
						Hex4 HexNAc4 Fuc								
						Hex4 HexNAc3 Fuc								
						Hex3 HexNAc3 Fuc								

Sites of glycosylation are listed as sequential numbers (N1-24), as sequential residue numbering with respect to the BaL sequence and with respect to HXB2CG (see Fig. 5). The theoretical tryptic peptide with the consensus site highlighted is also listed, with sub digest peptides used in further analyses utilising additional digestions detailed in Supplementary Tables S1-15. The observed glycosylation is summarised for each individual site (“Man” indicates an oligomannose structure of composition Hex_5-9_HexNAc_2_) with the specific glycomic and glycoproteomic experimental evidence used per site indicated. Supplementary Tables S1-15 provide detailed assignments. The abbreviations used are–RCM (reduced and carboxymethylated), T (trypsin), C (Chymotrypsin), G (Glu-C), PNGase (peptide-N-glycosidase), Man (Mannose), Hex (Hexose), HexNAc (N-acetylhexosamine), NeuAc (N-acetylneuraminic acid), Fuc (Fucose).
