# Supplementary material for: Mapping the complete glycoproteome of virion-derived HIV-1 gp120 provides insights into broadly neutralizing antibody binding
Source: Sci Rep. 2016 Sep 8;6:32956. doi: 10.1038/srep32956 (PMC5015092; doi:10.1038/srep32956)
Supplement: Supplementary Information [file srep32956-s1.pdf]

Mapping the complete glycoproteome of virion-derived HIV-1 gp120 provides insights into broadly neutralizing antibody binding

Maria Panico[1], Laura Bouché[1], Daniel Binet[2], Michael-John O'Connor[2], Dinah Rahman[1], Poh-Choo Pang[1], Kevin Canis[1#], Simon J. North[1], Ronald C. Desrosiers[3], Elena Chertova[4], Brandon F. Keele[4], Julian Bess, Jr[4], Jeffrey D. Lifson[4], Stuart M. Haslam[1], Anne Dell*[1], Howard R. Morris[1,2]

[1]Department of Life Sciences, Imperial College London, South Kensington Campus, London, SW7 2AZ, UK

[2]BioPharmaSpec, Suite 3.1 Lido Medical Centre, St. Saviours Road, Jersey, JE2 7LA, UK

[3] Department of Pathology, University of Miami, Miami, Florida, 33136, USA

[4]AIDS and Cancer Virus Program, Leidos Biomedical Research, Inc., Frederick National Laboratory, Frederick, Maryland, 21702, USA

#Current Address: SGS M-Scan SA, Geneva, Switzerland

*Correspondence should be addressed to Anne Dell (a.dell@imperial.ac.uk)

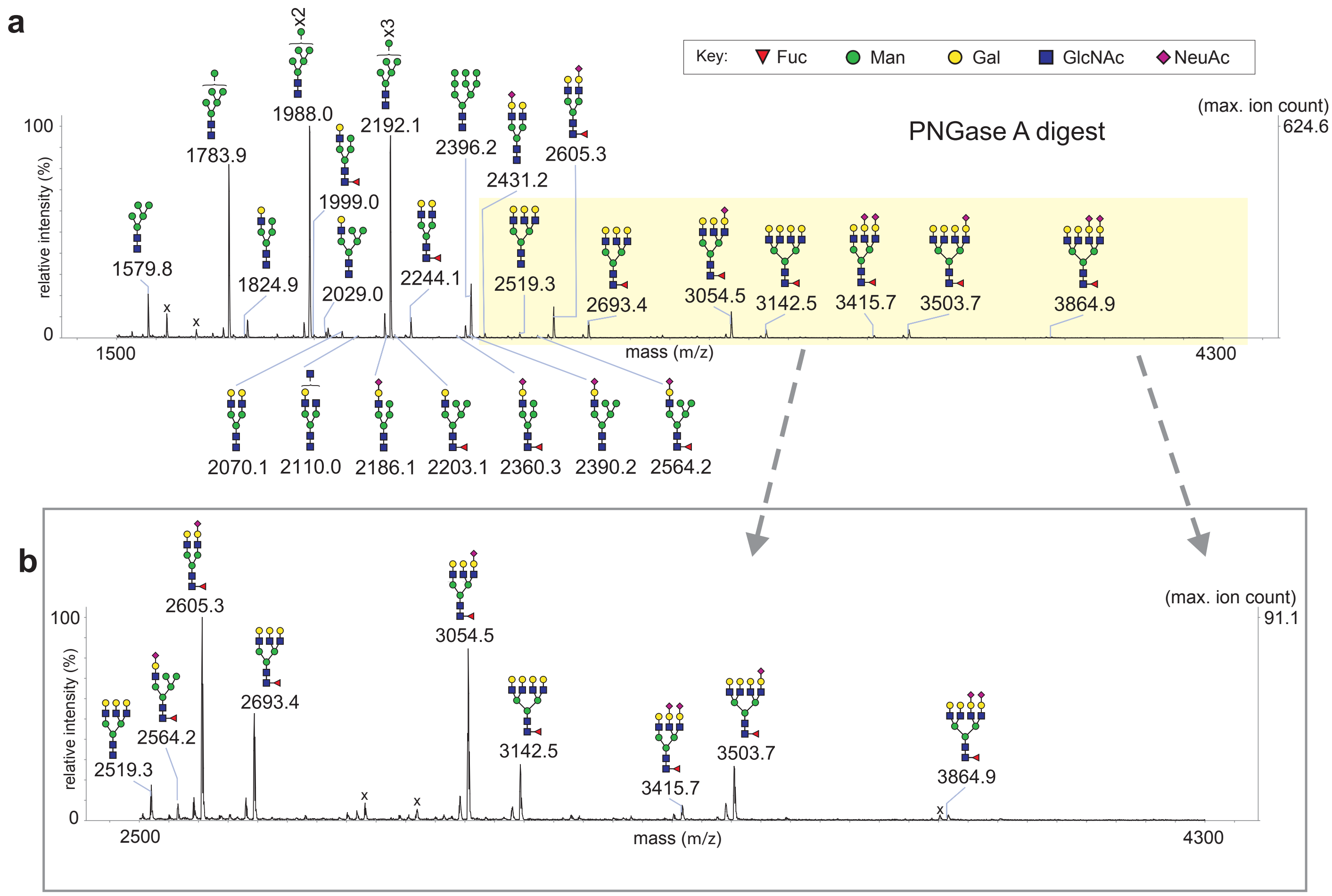

Supplementary Figure S1. N-glycome of HIV-1 Ba-L gp120. MALDI TOF mass spectrum of N-glycans released after PNGase A digest **(a)** full range spectrum m/z 1500-4300, **(b)** expanded region (m/z 2500-4300), highlighted in the full range mass spectrum. Signal abundance (relative intensity) is normalised to the most abundant ion (indicated on the right hand axis) of the specified mass range. Structural assignments were based on MS and MS/MS data and knowledge of N-glycan biosynthetic pathways. The satellite peaks near the major peaks are permethylation artefacts whilst the peaks which are labelled with an “x” are derived from known contaminants. Note that the N-glycan MS profile generated from PNGase A digest is very similar to the one generated from PNGase F digest.

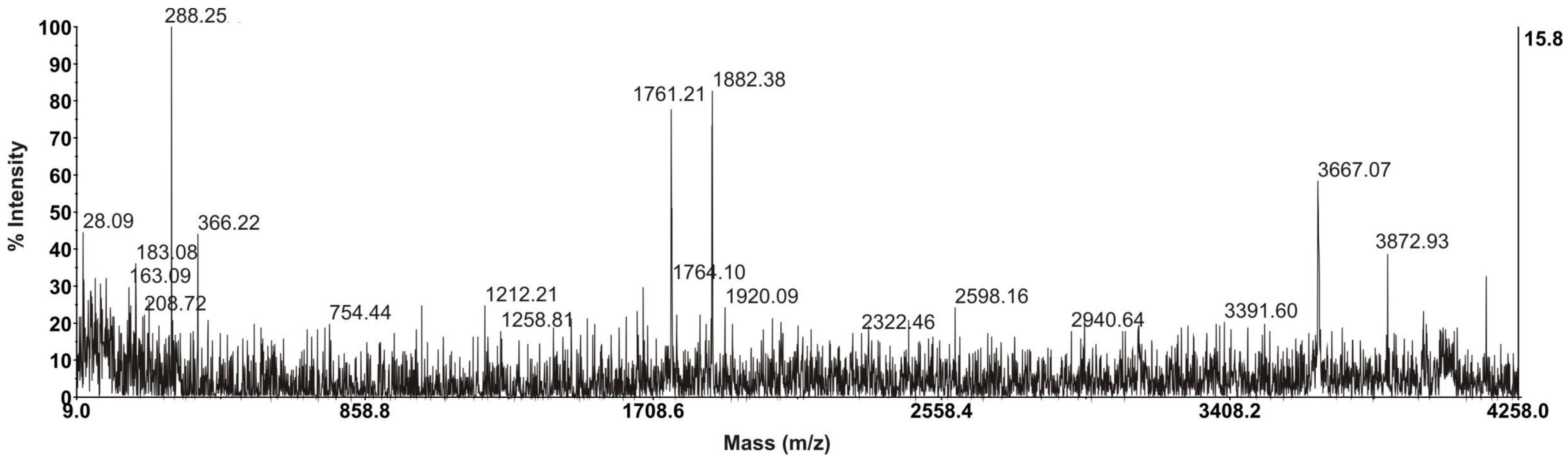

Supplementary Figure S2. Representative glycoproteomic data. MALDI-TOF/TOF spectrum of a relatively weak m/z 4030.9 signal which was observed together with related signals at m/z 4178, 3665.8 and 3811.8 in the MS data arising from the tryptic glycopeptide spanning Asn-129. Major fragment ions at m/z 1761.2 and m/z 1882.4 are assigned to the peptide plus a ring-cleavage of the first HexNAc, and to the peptide plus HexNAc itself, respectively, thus allowing the peptide mass to be determined as 1677 Da (determined more accurately in the electrospray experiment detailed in Supplementary Figures S3 and S4), corresponding to the sequence shown in Table 1. That in turn allows the complex carbohydrate composition to be assigned for the 4030.9 signal and the related signals in that group of glycoforms to be assigned similarly (see Table 1). The signal at m/z 288 is the y”2 fragment ion and the signal at m/z 3667 corresponds to a Hex.HexNAc loss from the non-reducing end of the glycopeptide glycan.

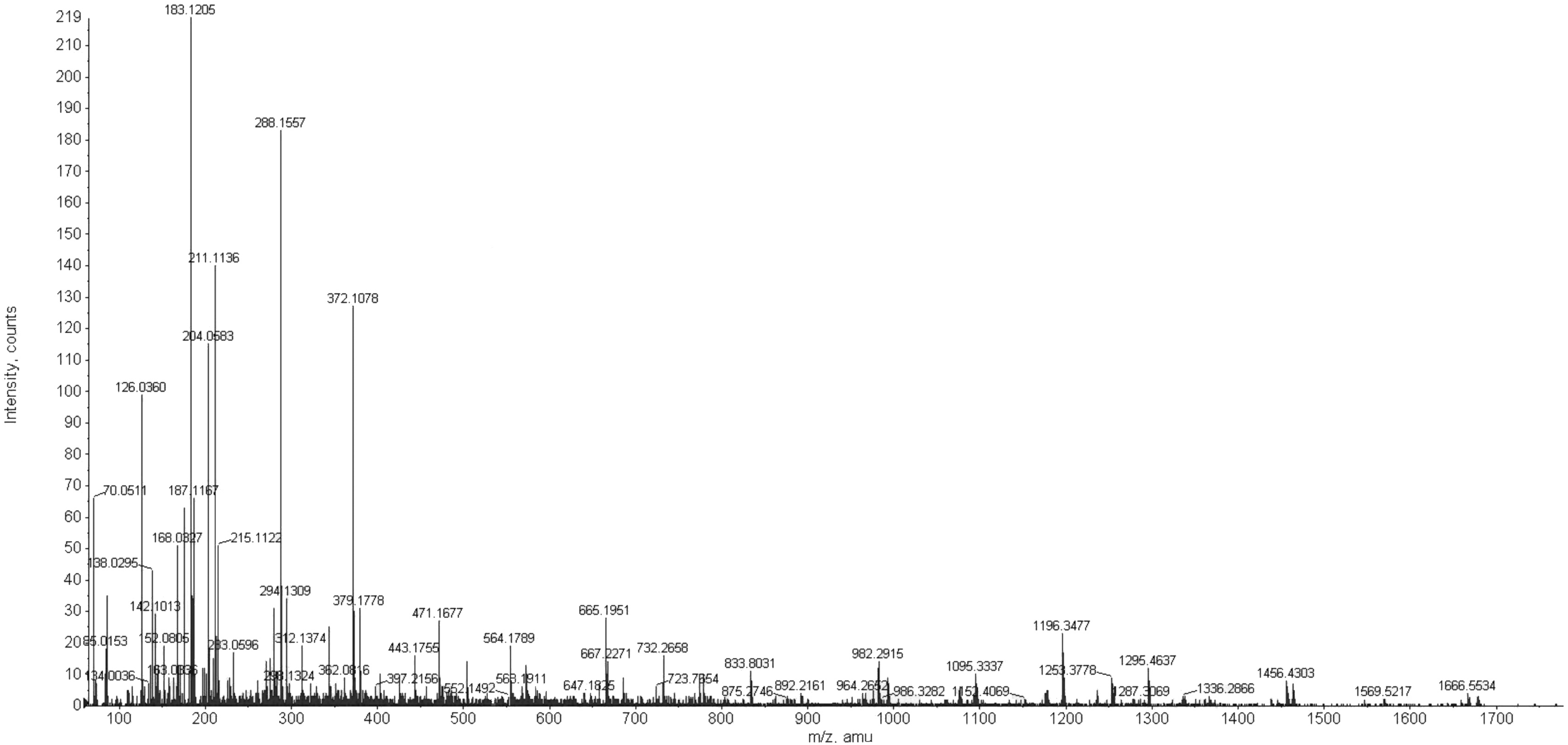

Supplementary Figure S3. Representative glycoproteomic data. ES MS/MS spectrum of m/z 940.8 corresponding to one of the glycoforms of the LTPLcmCVTLDcmCTDLR glycopeptide (the Asn-129 site) observed in the Tryptic/Endo-H data (reported in Supplementary Table S1-S15). The observed b and y” fragment ions are shown underlined in the peptide sequence depicted in Supplementary Figure S4. These data provide both confirmation of the peptide assignment and proof of the substitution position, because a HexNAc remains attached to Asn-129 in the y” ions from y”$_6$ upwards.

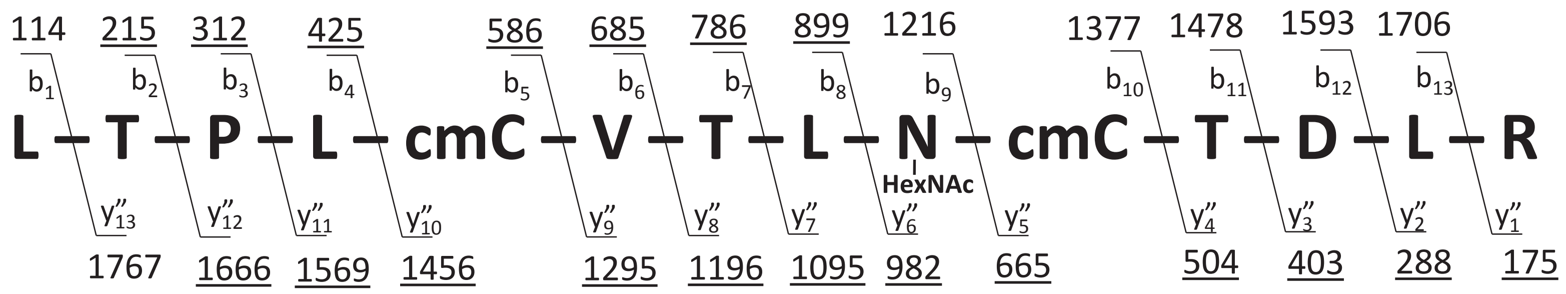

Supplementary Figure S4. Representative glycoproteomic data. Peptide sequence showing the observed b and y” fragment ions (underlined), corresponding to the ES MS/MS spectrum of m/z 940.8 depicted in Supplementary Figure S3.

These data provide both confirmation of the peptide assignment and proof of the substitution position, because a HexNAc remains attached to Asn-129 in the y” ions from y”6 upwards.

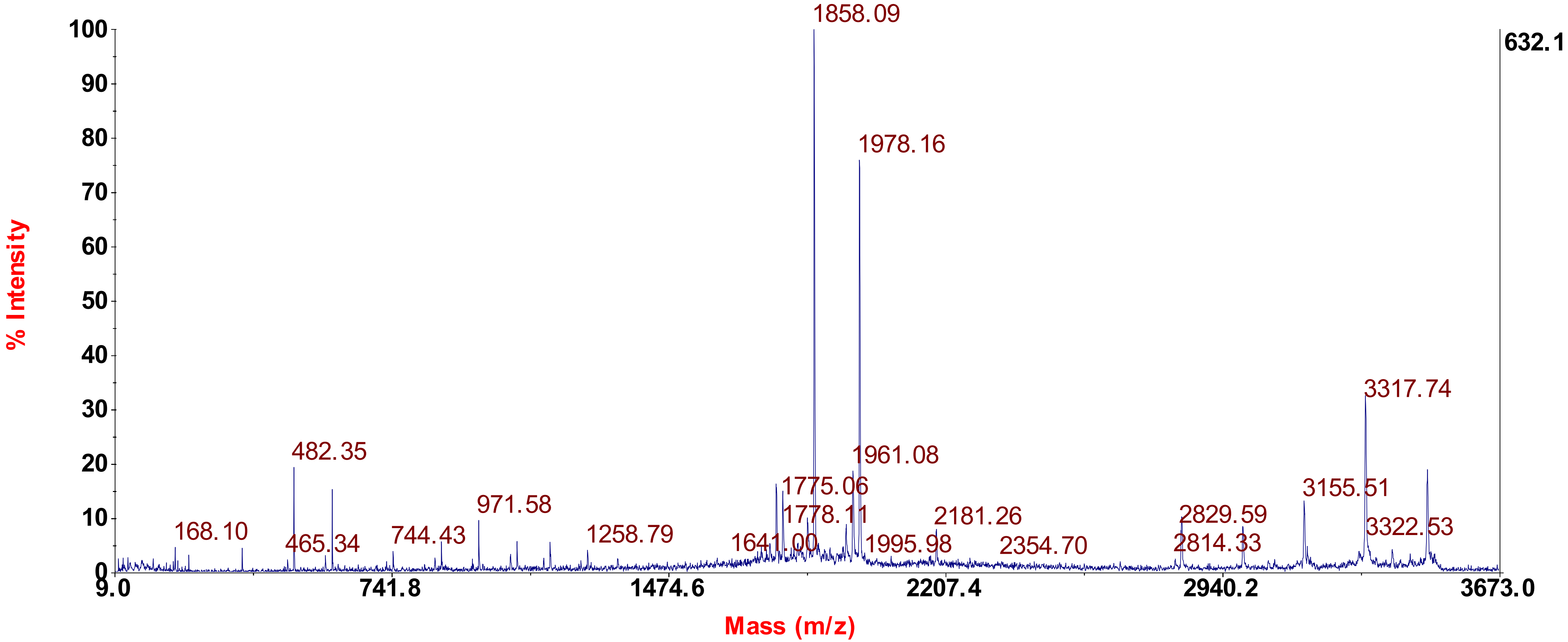

Supplementary Figure S5. Representative glycoproteomic data. Supplementary Figures S5 and S6 show MALDI-MS/MS spectra obtained on two "subdigest" tryptic peptides corresponding to partial cleavage between arginine and proline in the peptide spanning the two glycosylation sites at Asn-244 and Asn-265 (see Supplementary Tables S1-S15). The MALDI MS/MS data for these respective N- and C-terminal peptides bearing $HexNAc_2Hex_8$ (m/z 3477.5) and $HexNAc_2Hex_9$ (m/z 4131.1) are shown in Supplementary Figures S5 and S6, respectively. As in Supplementary Figure S2, the characteristic pairs of ions separated by 120 u, corresponding to the peptide plus a ring-cleavage of the first HexNAc, and to the peptide plus HexNAc itself, provide unambiguous evidence for the respective peptide masses in the fragmentation data, allowing calculation and assignment of the oligomannose glycans, supported overall by peptide fragment y" ions and, at high mass, by consecutive hexose losses from the non-reducing end of the glycan on the glycopeptide.

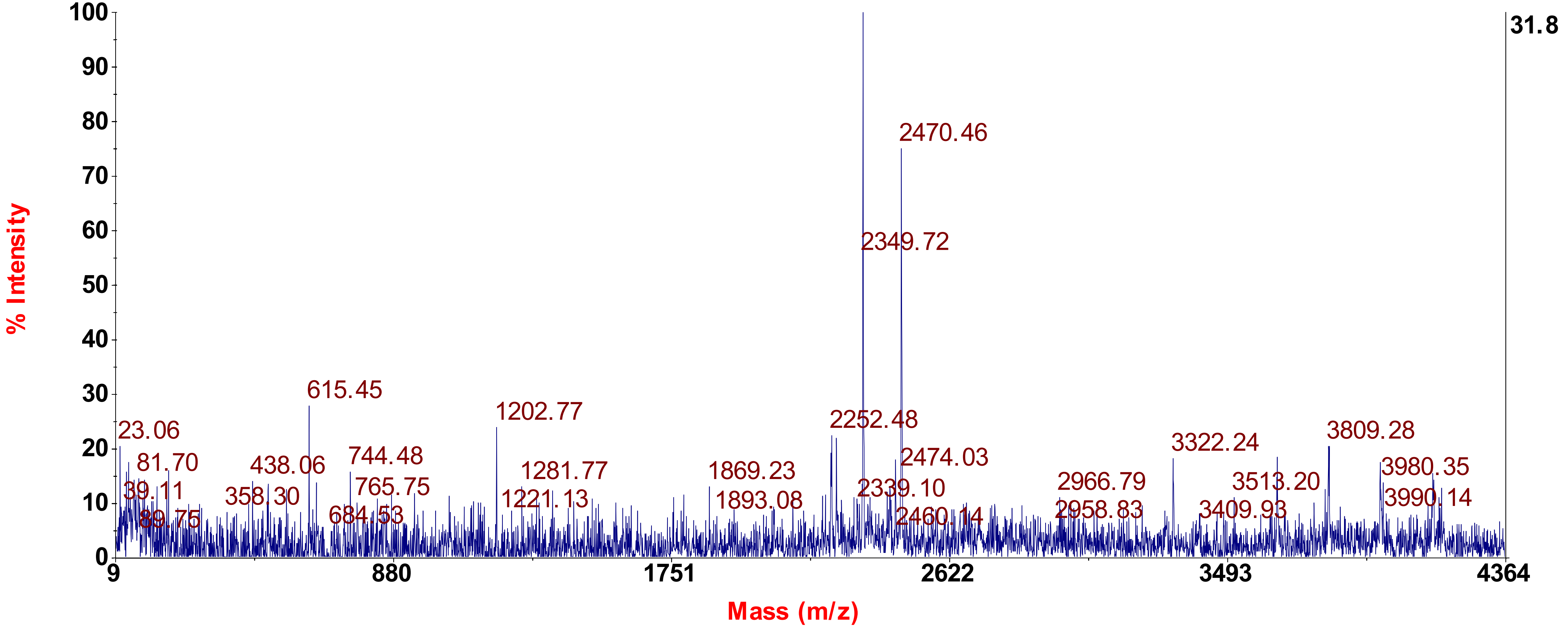

Supplementary Figure S6. Representative glycoproteomic data. Supplementary Figures S5 and S6 show MALDI-MS/MS spectra obtained on two "subdigest" tryptic peptides corresponding to partial cleavage between arginine and proline in the peptide spanning the two glycosylation sites at Asn-244 and Asn-265 (see Supplementary Tables S1-S15). The MALDI MS/MS data for these respective N- and C-terminal peptides bearing $HexNAc_2Hex_8$ (m/z 3477.5) and $HexNAc_2Hex_9$ (m/z 4131.1) are shown in Supplementary Figures S5 and S6, respectively. As in Supplementary Figure S2, the characteristic pairs of ions separated by 120 u, corresponding to the peptide plus a ring-cleavage of the first HexNAc, and to the peptide plus HexNAc itself, provide unambiguous evidence for the respective peptide masses in the fragmentation data, allowing calculation and assignment of the oligomannose glycans, supported overall again by peptide fragment y" ions and here by consecutive hexose losses from the non-reducing end of the glycan on the respective glycopeptides.
